# Determinants of under-five mortality in the high mortality regions of Ethiopia: mixed-effect logistic regression analysis

**DOI:** 10.1186/s13690-021-00578-4

**Published:** 2021-04-23

**Authors:** Misganaw Gebrie Worku, Achamyeleh Birhanu Teshale, Getayeneh Antehunegn Tesema

**Affiliations:** 1 Department of Human Anatomy, College of Medicine and Health Science, School of Medicine, University of Gondar, Gondar, Ethiopia; 2 Department of Epidemiology and Biostatistics, Institute of Public Health, College of Medicine and Health Sciences, University of Gondar, Gondar, Ethiopia

**Keywords:** Ethiopia, Under-five mortality, Mixed-effect analysis

## Abstract

**Background:**

Even though the global under-five mortality rate substantially decreased over time, Sub-Saharan African (SSA) countries including Ethiopia continue to share the huge burden of under-five mortality. Ethiopia showed a substantial reduction in under-five mortality over time but the rate of reduction has varied across regions. Therefore, this study aimed to investigate determinants of under-five mortality in the high mortality regions of Ethiopia.

**Methods:**

A secondary data analysis was done based on the 2016 Ethiopian Demographic and Health Survey (EDHS) data. A total weighted sample of 3446 live births were included for this study. For the determinants of under-five mortality, mixed-effect logistic regression was fitted. The Intra-Class Correlation Coefficient (ICC), and Median Odds Ratio (MOR) were done to assess the presence of a significant clustering effect. The standard binary logistic regression and the mixed-effect logistic regression model were fitted and deviance (−2LL) was used for model comparison as the models were nested models. Variables with a *p*-value less than 0.2 in the bi-variable mixed-effect binary logistic regression analysis were considered for the multivariable analysis. In the multivariable mixed-effect logistic regression analysis, the Adjusted Odds Ratio (AOR) with the 95% Confidence Interval (CI) were reported to declare the statistical significance and strength of association of under-five mortality and the determinant factors.

**Results:**

Overall, the under-five mortality rate in the high mortality regions of Ethiopia was 74 per 1000 live births and it was highest among twin births (262 per 1000 live births). In the multivariable mixed-effect logistic regression analysis, being having 6 and above births (AOR = 3.66, 95% CI: 1.55, 8.67), preceding birth interval of 2–3 years (AOR = 0.57, 95% CI: 0.41, 0.81) and above 3 years (AOR = 0.35, 95% CI: 0.22, 0.55), being twin (AOR = 5.12, 95% CI: 2.28, 11.46), and being having antenatal care (ANC) visit during pregnancy (AOR = 0.27, 95% CI: 0.16, 0.45) were significant determinants of under-five mortality.

**Conclusion:**

In this study, under-five mortality rate was highest in high mortality regions of Ethiopia. Parity, ANC visit, preceding birth interval, and multiple births were significant predictors of under-five mortality. Therefore, public health interventions that increase maternal health service utilization such as ANC and family planning service utilization to increase birth interval are needed to reduce under-five mortality among these regions of Ethiopia.

## Background

Under-five mortality is the death of a child before reaching the age of 5 years [1]. The first 5 years of life are the most crucial years for the physical and intellectual development of children [1].

Even though there is a worldwide decrement in under-five mortality, from 5.9 million deaths in 2015 to 5.3 million in 2018, still there is a high mortality rate in African countries (81 per 1000 live births) including Ethiopia, which is around 7 times higher than in the European region [1, 2]. In Africa, under-five mortality contributes for 14% of the global burden of child mortality and in sub-Saharan Africa, it accounts for nearly 50% of child mortality, while it accounts for just 11% of the world’s population [3]. Sub-Saharan Africa countries had the highest under-five mortality with 1 child in every 13 live birth dying before 5 years of age, which is 15 times higher than in high-income countries and half of all these deaths in 2018 occurred in five countries; India, Nigeria, Pakistan, Ethiopia, and the Democratic Republic of the Congo [3, 4].

Under-five child mortality in Ethiopia is one of the biggest and most difficult problems that should be given emphasis [4]. Millennium development goal (MDG) 4 was aimed at reducing under-five mortality by two-thirds and this was unfinished agenda for most of the middle and lower-income countries [5]. With the current rates of child mortality in Ethiopia achieving the sustainable development goal (SDG), which is reducing under-five mortality to 55 deaths per 1000 live births by 2030 is difficult and studies revealed that there is a significant regional variation in under-five mortality [4].

That is, in some regions of Ethiopia, under-five mortality is still devastating and according to the 2016 Ethiopian Demographic and Health Survey (EDHS) report, under-five mortality per 1000 live birth in afar, Benishangul-Gumuz, and Somali were 125, 98, and 94, respectively [6].

Evidence from different literature showed that low level of maternal education, unsafe drinking water and sanitation, low family income, birth interval, short breastfeeding time, and place of delivery are among the determinants of under-five mortality [4, 7–19].

Even though Ethiopia is working on the reduction of under-five mortality, through improving coverage, quality and use of skilled care, essential newborn care, and management of preterm and low birth weight, still it is very high in the regions of Afar, Somalia, and Benishangul-Gumuz [5]. Therefore, this study aimed to explore the determinants of under-five mortality in these high-risk regions of Ethiopia. This study might give insight for health professionals, policymakers, and the community in general for appropriate intervention to reduce under-five mortality.

## Methods

### Data source

Secondary data analysis was conducted based on the EDHS 2016, which is the fourth survey conducted nationally from January 18 to June 27, 2016. The EDHS employed a two-stage stratified sampling technique to select respondents. In the first stage, 645 enumeration areas (EAs) (202 in urban areas and 443 in rural areas) were selected. In the second stage, a fixed number of 28 households per cluster was selected, with an equal probability systematic selection from the newly created household list. The detailed sampling procedure was presented in the full EDHS 2016 report [6].

**Fig. 1:**
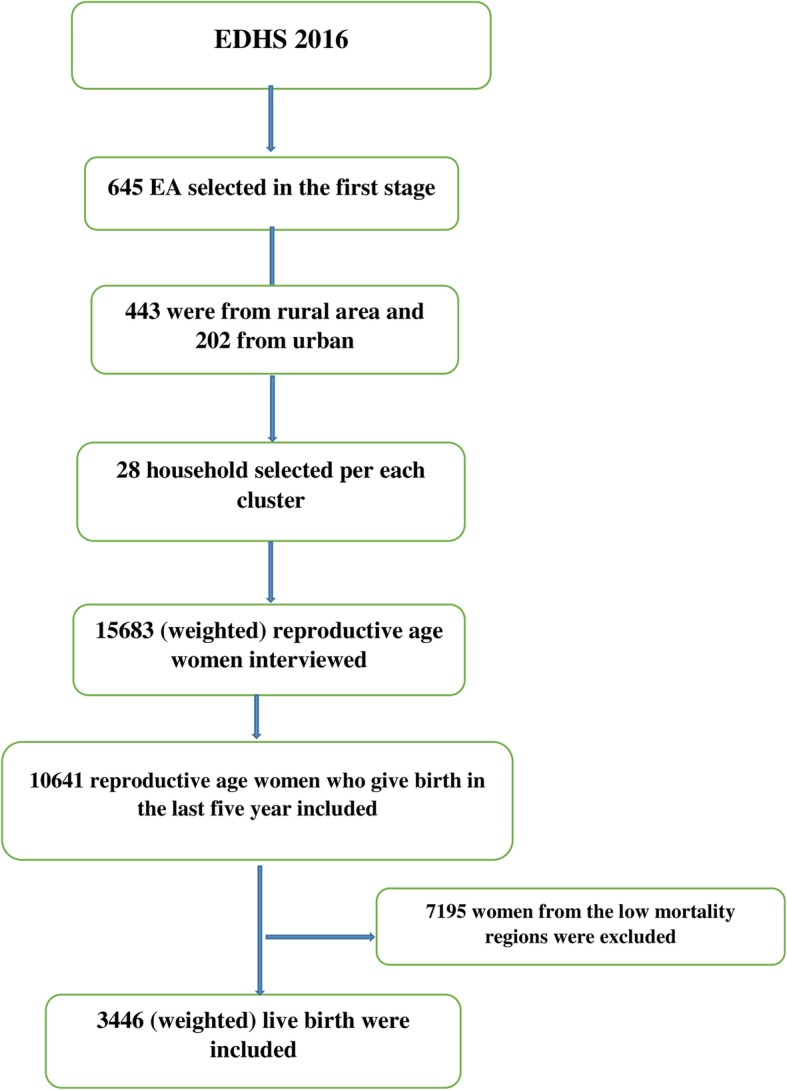
Flow diagram showing the sampling

Afar, Somali, and Benishangul-Gumuz regions were selected since based on the 2016 EDHS report, these regions had the highest rates of under-five mortality. A total weighted sample of 3446 women with a live birth in the 5 years preceding the survey were included for this study. For women who had two or more live births in the 5 years preceding the survey, the last birth was taken (Fig. 1).

### Study variables

The outcome variable was the death of under-five children, which is a dichotomous variable coded as “0” if the child is alive and “1” if the child is died.

The independent variables were: proximal factors (sex of a child and multiple births), intermediate factors (mother’s level of education, birth order, preceding birth interval, parity, ANC visit, respondent current age, and place of delivery), and distant factors (household wealth index, source of drinking water, sex of household head, place of residency, and presence of toilet facility) (Table 1).

**Table 1:** Description and measurement of independent variables

Independent variables and their description/categorization	
Respondent age	Current age of the mother and re-coded in to two categories with values of “0” for < 20, “1” for 20–24, “2” for 25–29, “3” for 30–36 and “4” for ≥35 years.
Religion	Religion of the mother that is re-coded in to three categories, “0” for Muslim, “1” for Orthodox and “2” for other religious groups (combining protestant, catholic, traditional, and the other religious categories).
Wealth status	It is the household wealth status and in the EDHS data it was created using principal components analysis and coded as “poorest”, “poorer”, “middle”, “richer”, and “richest”. For this study, we recoded it in to three categories as “poor” (includes the poorest and the poorer categories), “middle”, and “rich” (includes the richer and the richest categories)
Parity	It is the total number of children a women had and re-coded in to three categories with a value of “0” for 1–3, “1” for 4–6, and “2” for > 6 children.
Birth order	Recoded in to four categories with a value of “0” for first birth order and “1” for 2-3rd order, “2” for 4-5th, and “3” for 6th and above birth order.
Toilet facility	The variable type of toilet facility is recoded as “0” for pit latrine (flush to piped sewer system, flush to septic tank, flush to pit latrine, flush, don’t know where, ventilated improved pit latrine, pit latrine with slab, and pit latrine without slab/open pit), and “1” for no pit toilet latrine (no facility/bush/field, composting toilet, and others)
Educational status	This is the minimum educational level the mother achieved and coded in to two groups with a value of “0” for no education and “1” for primary education and above.
Sex of household	The variable sex of household head was corded as male and female in the dataset and we used without change.
Water source	Recoded in to two categories with value of “0” for “pipe (piped into dwelling, piped to yard/plot, piped to neighbor, and public tap/standpipe) and” “1” for other source of water.
Preceding birth interval	The variable preceding birth interval is recoded as “0” for preceding birth interval of ≤2 years, “1” for preceding birth interval of 2–3 years, and “2” for preceding birth interval of > 3 years.
Place of delivery	The variable place of delivery was recoded as “0” for home delivery and “1” for delivery in the health facility.
Sex of child	The sex of the child recoded as “1” for male and “2” for female in the EDHS and used without recoding.
Birth type	The variable birth type was recoded in to two categories as “0” for single birth and “1” for multiple birth.
ANC visit	The variable ANC visit was recoded in to two categories, “0” for woman who had no any ANC visit during their pregnancy and “1” for woman who had at least one ANC visit during their pregnancy.
Place residence	The variable place of residence was corded as rural and urban in the dataset and used without change for this study.
Region	The variable region was coded in to 11 categories in the dataset and we used the three (Afar, Somali, and Beni shangul).

### Data management and analysis

Stata 14 software was used for extracting, recoding, and analysis of data. The data were weighted using sampling weight, primary sampling unit, and strata before any statistical analysis to restore the representativeness of the data and to get a reliable estimate and standard error. Descriptive statistics were done using frequencies and percentages. The EDHS data has a hierarchical structure; this violates the independent assumptions of a standard logistic regression model. Therefore, to assess the determinant factors, the mixed-effects logistic regression model with the Generalized Linear Mixed Model (GLMM) framework was used.

$$ \log\ \left[\uppi \mathrm{ij}/\left(1-\uppi \mathrm{ij}\right)\right]=\upbeta 0+\upbeta 1\mathrm{X}2\mathrm{ij}+\upbeta 2\mathrm{X}3\mathrm{ij}+\upbeta 3\mathrm{X}2\mathrm{ij}+\cdots +\upbeta \mathrm{nXnij}+\cup 0\mathrm{i}+\upepsilon \mathrm{ij} $$

πij = is probability of under-five mortality

1-πij = is probability of surviving in the first 5 years of birth

β0 = is log odds of the fixed intercept

X1ij… Xnij = are independent variables of individuals and community’s level

β1… βn are effect sizes of individual levels and community-level coefficients

uOj are random errors at cluster level

ϵij random error at individual level estimated by π^2^/3

Multicollinearity between independent variables was assessed using Variance Inflation Factor (VIF) and each of the independent variables and the mean VIF was less than 5. Intraclass Correlation Coefficient (ICC) and Median Odds Ratio (MOR) were checked to assess whether there was clustering and deviance was used for model comparison. Both bivariable and multivariable mixed-effect logistic regression were done. At the bivariable analysis variables with a *p*-value < of 0.2 were considered for multivariable analysis and variables with a *P*-value of < 0.05 in the multivariable analysis were considered as determinants of under-five mortality.

## Results

### Sociodemographic characteristics of study participants

A total weighted sample of 3446 was included in this study. A majority (80.9%) of the mothers were without formal education. Approximately, 87% of study participants were from rural areas and 43.67% were from the Somali region. Most (85.95%) of respondents were followers of the Muslim religion. About 19.38% of respondents were from households with safe drinking water supplies and the majority (58.5%) of participants were from households with no toilet facility. Concerning wealth status, 76% of respondents were from households with a poor wealth status. Most (82.3%) of mothers gave their birth at home and 70.3% of mothers did not have ANC visit during their last pregnancy. Regarding birth order, 30% of mothers had a birth order greater than six and 41% of mothers gave birth with a preceding birth interval less than 2 years. Moreover, 52.3% of children were males and the great majority (98.8%) were singletons (Table 2).

**Table 2:** Descriptive characteristics of the respondents in Ethiopia, 2016 (*N* = 3446)

Variable	weighted frequency (%)
Regions	
Afar	1062 (30.8%)
Somali	1505 (43.6%)
Benishangul-gumuz	879 (25.5%)
Respondent current age	
< 20	154 (4.5%)
20–24	793 (23%)
25–29	1025 (29.7%)
30–34	706 (20.5%)
≥ 35	768 (22.3%)
Religion	
Muslim	2962 (86%)
Orthodox	256 (7.4%)
Others	228 (6.6%)
Wealth index	
poor	2618 (76%)
Middle	251 (7.3%)
Rich	577 (16.7%)
Residence	
Urban	445 (13%)
Rural	3001 (87%)
Source water	
Pipe	668 (19.4%)
Other	2778 (80.6%)
Toilet facility	
Yes	1419 (41.2%)
No	2027 (58.8%)
Sex of household	
Male	2432 (70.6%)
Female	1014 (29.4%)
Mother education	
No education	22,760 (80%)
Primary and above	686 (20%)
Birth order	
1st	595 (17.3%)
2–3	999 (29%)
4–5	830 (24%)
6 and above	1022 (29.7%)
Parity	
1–3	1311 (38%)
4–6	1267 (36.8%)
> 6	868 (25.2%)
Preceding birth interval	
≤ 2 years	1160 (40.7%)
2–3 years	922 (32.4%)
Above3 years	765 (26.9%)
Place of delivery	
Home	2837 (82.3%)
Health facility	609 (17.7%)
Child characteristics	
sex	
Male	1803 (52.3%)
Female	1643 (47.7%)
Birth type	
Single	3404 (98.8%)
Multiple	42 (1.2%)
ANC	
Yes	1023 (29.7%)
No	2423 (70.3%)

### Prevalence of under-five mortality in the high-risks region of Ethiopia

Two hundred fifty-seven children (7.46, 95%CI; 6.6, 8.3%) died before 5 years of age from total live birth with a mortality rate of 74 per 1000 live births. Among mothers with no ANC visit during their last pregnancy, there is higher under-five mortality (95 deaths per 1000 live births) relative to mothers with ANC visits (26 death per 1000 live birth). In multiple births, the rate of under-five mortality was higher (27 deaths per 1000 live births) than in singletons (8 deaths per 1000 live births). The under-five mortality rate is also differed with wealth status, falling from 80 deaths to 53 deaths per 1000 live births in poor vs rich households. In rural and urban areas, the under-five mortality rate was 78 and 47 deaths per 1000 live births, respectively. In households with untreated water sources, the under-five mortality rate was higher (78 deaths per 1000 live births) than in those with a safe water source (60 deaths per 1000). Among households with no standard toilet facility, under-five mortality was higher (79 deaths per 1000 live births) compared to those with a standard toilet facility (67 deaths per 1000 live births) (Table 3).

**Table 3:** The prevalence of under-five mortality in Ethiopia, 2016 (*N* = 3446)

Variables	Total	No of children died	Under-five mortality rate per 1000
Respondent current age			
< 20	154	14	91
20–24	793	56	71
25–29	1025	68	66
30–34	706	50	71
> =35	768	69	90
Religion			
Muslim	2962	222	75
Orthodox	256	16	62
Others	228	19	83
Sex			
Male	1803	137	76
Female	1643	120	73
ANC			
Yes	1023	27	26
No	2423	230	95
Birth type			
Single	3404	246	72
Multiple	42	11	262
Mother education			
No education	2760	214	77
Primary and above	686	43	63
Birth order			
1st	595	57	96
2–3	999	60	60
4th and above	1852	140	75
Parity			
1–3	1311	86	66
4–6	1267	85	67
> 6	868	86	99
Wealth index			
Poor	2618	212	81
Middle	251	14	56
Rich	577	31	54
Residency			
Urban	445	21	47
Rural	3001	236	79
Place of delivery			
Home	2837	223	79
Health facility	609	34	56
Source water			
Pipe	668	40	60
Other	2778	217	78
Toilet facility			
Yes	1419	96	68
No	2027	161	79
Regions			
Afar	1062	90	85
Somali	1505	103	68
Benishangul-Gumuz	879	64	73

### Determinants of under-five mortality in the high-risks region of Ethiopia

#### Model comparison/random effect analysis

Log-likelihood Ratio (LLR) test and deviance were checked, and the mixed effect logistic regression model was chosen since it had the smallest value of deviance. Furthermore, the ICC value was 0.14 and this revealed about 14% of the variability of under-five mortality was attributed due to the difference between communities/clusters. The median odds ratio was 1.49, indicating that if we randomly select one child from a higher-risk cluster (cluster with higher under-five mortality) the odds of under-five mortality is 1.49 times compared to those from a lower-risk cluster. Moreover, the Log-likelihood ratio test was significant (*X*^2^ value = 4.63, *P* value = 0.0157). These all informed us to choose a mixed-effect logistic regression model (GLMM) over the basic model (Table 4).

**Table 4:** Model comparison and fitness for the assessment of determinants of under-five mortality

Model comparison	The standard logistic regression model	Mixed effect logistic regression model
Log-likelihood	− 668.0	− 667.29
Deviance	1336	1334.58
Intraclass correlation(ICC) and median odds ratio(MOR) of the mixed effect model (Final model)		
ICC	0.14	
MOR	1.49	

#### Fixed effects analysis

Determinants of under-five mortality in the high-risk regions of Ethiopia were analyzed using a mixed-effect logistic regression model. Variables with a *p*-value < 0.2 in the bivariable analysis were eligible for the multivariable analysis. In the multivariable analysis, parity, preceding birth interval, birth type, and ANC visit were significant determinants of under-five mortality. The odds of under-five mortality was 3.66 (AOR = 3.66: 95%CI; 1.55, 8.68) times higher among mothers with parity of greater than six compared with those mothers having children of three and less in the family. Mothers with preceding birth intervals of two to 3 years and above 3 years had 43% (AOR = 0.57: 95%CI; 0.41, 0.81) and 65% (AOR = 0.35: 95% CI; 0.22, 0.55) lower odds of under-five mortality as compared to mothers with preceding birth interval of fewer than 2 years. Being multiple births had 5.1 (AOR = 5.1: 95%CI; 2.28, 11.46) times higher odds of under-five mortality compared to a single birth. Moreover, there was a 63% (AOR = 0.27 95%CI; 0.16, 0.45) lower odds of under-five mortality among mothers with ANC visit during their last pregnancy compared to their counterparts (Table 5).

**Table 5:** Multivariable mixed effect analysis for assessing the determinants of under-five mortality in high-risk regions of Ethiopia, 2016

Variables	COR (95% CI)	AOR (95% CI)
**Parity**		
1–3	1	1
4–6	1.01 (0.73, 1.38)	1.72 (0.99, 2.99)
> 6	1.59 (1.15, 2.19)	3.66 (1.55, 8.67)
**Wealth status**		
Poor	1	1
Middle	0.67 (0.38, 1.19)	0.88 (0.45, 1.7)
Rich	0.63 (0.42, 0.96)	0.67 (0.35, 1.3)
**Birth interval**		
2–3 years	0.55 (0.39, 0.77)	0.57 (0.41, 0.81)
above 3 years	0.32 (0.205,0.49)	0.35 (0.22, 0.55)
**Residence**		
Urban	1	1
Rural	1.71 (1.04, 2.8)	1.15 (0.52, 2.56)
**Type of birth**		
Single	1	1
multiple	4.42 (2.15, 9.06)	5.12 (2.28, 11.46)
**Source of drinking water**		
Protected	1	1
Unprotected	1.37 (0.94, 1.99)	1.06 (0.67, 1.69)
**Sex of household head**		
Male	1	1
Female	0.77 (0.57, 1.05)	0.84 (0.58, 1.2)
**Place of delivery**		
Home	1	1
health facility	0.69 (0.47, 1.02)	1.07 (0.63, 1.8)
**Mother education**		
No education	1	1
primary and above	0.8 (0.57, 1.14)	1.37 (0.85, 2.22)
**ANC visit**		
No	1	1
Yes	0.25 (0.17, 0.38)	0.27 (0.16, 0.45)

## Discussion

The SDG is aimed at reducing preventable deaths of newborns and children under the age of five [9, 16]. In this study, 7.46% (95%CI; 6.6, 8.3%) of under-five children had died before celebrating their fifth birthday with a mortality rate of 74 per 1000 live birth. This finding was relatively higher than another study done in Ethiopia [10] and lower than other studies conducted in Ethiopia [2, 11]. The disparity may be attributed to variations in sample size and study setting, as the sample size in our study was relatively small compared to others. Besides, the discrepancy or the higher prevalence found in this study might be due to the poor socioeconomic status of the participants in these high-risk regions of Ethiopia [10].

The multivariable mixed-effects analysis showed that type of birth, ANC visit, preceding birth interval, and parity had significant associations with under-five mortality.

The odds of under-five mortality among multiple births were higher as compared to singleton births and this is consistent with different studies conducted in Ethiopia [2, 3, 11, 12]. This might be because multiple births can lead to growth retardation and prematurity, which are the main risk factor for under-five mortality [13]. In addition, being twins or triplets might result in under-nutrition because of inadequate breast milk as well as infections due to inappropriate formula feeding and feeding of cow’s milk.

In the present study, under-five mortality increases as parity increases. This is consistent with a study carried out in Ethiopia [2] but in contrast to other studies [7, 14]. This might be because mothers with higher parity might be busy caring for many children in the family and may give limited attention to the health of their last child. Besides, mothers with higher parity had enough number of children so they might be careless in seeking appropriate care from the health facility for this child [17]. Additionally, in this study, there were higher odds of under-five mortality among children born from mothers with a previous birth interval of fewer than 2 years. This finding is in line with other studies [2–4]. This might be as a result of short birth interval might lead to premature birth and limited family resources because of the increased number of children in the family [11, 16, 19]. Also, mothers with short preceding birth interval might be exposed to malnutrition and this, in turn, affect her child in getting appropriate nutrients from the mother and might expose the child to malnutrition and infections, which finally ends up with death.

Moreover, children of women who had ANC visits during pregnancy had a lower risk of mortality relative to those of mothers with no ANC visits. This finding is consistent with the study conducted in Ethiopia [4] but in contrast with other studies [3, 15]. This might be because mothers with appropriate utilization of maternal health services such as ANC visits might induce appropriate information for the mother regarding the health of the newborn and appropriate feeding practices to prevent infections [18]. In addition, mothers with ANC visits might have a probability of taking postnatal care that is crucial for searching for childhood health problems and taking appropriate intervention timely.

### Strength and limitation of the study

This study was based on nationally representative data with a large sample size. Besides, it was based on an appropriate statistical approach (mixed-effect analysis) to accommodate the community or cluster level variability of under-five mortality. Moreover, since it is based on the national survey data the study has the potential to give insight for policy-makers and program planners to design appropriate intervention strategies both at national and regional levels. However, this study had limitations in that the EDHS is mostly based on respondents’ self-report and might have the possibility of recall bias. Besides, since the study was based on the information available in the EDHS data, there might be residual confounding factors.

## Conclusion

In the high-risk region of Ethiopia the prevalence of under-five mortality is high. Under-five mortality in these regions was significantly associated with preceding birth interval, type of birth, parity, and ANC visits. Thus, special emphasis should be given to those mothers with no ANC visit, preceding birth interval of fewer than 2 years, mothers with higher parity, and multiple births.

## Data Availability

The data set is available online and any one can access it from www.measuredhs.com.

## Abbreviations

ANC: Antenatal care
CI: Confidence interval
EA: Enumeration area
EDHS: Ethiopian demographic health survey
GLMM: Generalized linear mixed model
ICC: Intraclass Correlation Coefficient
LLR: Likelihood ratio
MDG: Millennium development goal
SDG: Sustainable development goals
WHO: World Health Organization
